# Combination therapy with polymyxin B and netropsin against clinical isolates of multidrug-resistant *Acinetobacter baumannii*

**DOI:** 10.1038/srep28168

**Published:** 2016-06-16

**Authors:** Joon-hui Chung, Abhayprasad Bhat, Chang-Jin Kim, Dongeun Yong, Choong-Min Ryu

**Affiliations:** 1 Super-bacteria Research Center, Korea Research Institute of Bioscience and Biotechnology, Daejeon 34141, South Korea; 2 University of Science and Technology, Daejeon 34113, South Korea; 3 Industrial Bio-materials Research Center, Korea Research Institute of Bioscience and Biotechnology, Daejeon 34141, South Korea; 4 Department of Laboratory Medicine and Research Institute of Antimicrobial Resistance, Yonsei University College of Medicine, Seoul 03722, South Korea

## Abstract

Polymyxins are last-resort antibiotics for treating infections of Gram-negative bacteria. The recent emergence of polymyxin-resistant bacteria, however, urgently demands clinical optimisation of polymyxin use to minimise further evolution of resistance. In this study we developed a novel combination therapy using minimal concentrations of polymyxin B. After large-scale screening of *Streptomyces* secondary metabolites, we identified a reliable polymixin synergist and confirmed as netropsin using high-pressure liquid chromatography, nuclear magnetic resonance, and mass spectrometry followed by *in vitro* assays using various Gram-negative pathogenic bacteria. To evaluate the effectiveness of combining polymixin B and netropsin *in vivo*, we performed survival analysis on greater wax moth *Galleria mellonella* infected with colistin-resistant clinical *Acinetobacter baumannii* isolates as well as *Escherichia coli, Shigella flexineri, Salmonella typhimuruim,* and *Pseudomonas aeruginosa*. The survival of infected *G. mellonella* was significantly higher when treated with polymyxin B and netropsin in combination than when treated with polymyxin B or netropsin alone. We propose a netropsin combination therapy that minimises the use of polymyxin B when treating infections with multidrug resistant Gram-negative bacteria.

Life-threatening bacterial infections are a major concern in health care settings worldwide. Because of increase in the emergence of antibiotic resistant bacteria, medical practitioners must look to rarely used antibiotics for alternative treatment options^1^,^2^. For instance, bacteria such as *Acinetobacter baumannii* cause nosocomial infections in immunocompromised patients, which, until recently, could be effectively treated with carbapenems^3^. The recent emergence of carbapenem-resistant Gram-negative pathogenic bacteria, such as *A. baumannii*, *Escherichia coli*, *Pseudomonas aeruginosa*, and *Klebsiella pneumonia*, has led to the resurgence of polymyxins as last-resort antibiotic treatments^3^,^4^,^5^. Multidrug resistant (MDR) *A. baumannii* infections are associated with intensive care units and are implicated in ventilator associated pneumonia and urinary tract infections^4^. In the recent times MDR *A. baumannii* are reported to exhibit a variety of resistance mechanisms to most classes of antibiotics which demands immediate development of new therapies^6^.

Polymyxins are cataionic lipopeptide antibiotics produced by non-ribosomal peptide synthetase enzyme complexes and are bactericidal agents with a detergent-like mechanism of action^7^. Colistin (polymixin E) and polymixin B are the two important forms of polymixnis with clinical significance^8^. They have largely been used in topical form as otic and ophthalmic solutions and are also used in combination with numerous other antibiotics, such as carbapenems, efflux pump inhibitors, β-lactamase inhibitors, and wall teichoic acid synthesis inhibitors^7^,^8^,^9^,^10^. However, the use of polymyxins is limited by their nephrotoxic and neurotoxic effects and hence administered mostly to prevent rather than treat infections^11^,^12^. Furthermore, the pharmacokinetics and pharmacodynamics of polymyxin B remain poorly understood. In addition, there has been a surge in reports of infections caused by naturally-occurring, polymyxin-resistant bacteria, including *Providencia* spp.*, Proteus* spp.*, Morganella* spp., and *Serretia* spp.^4^ There is thus urgency to optimise the clinical use of polymixins by designing effective combination therapies^11^,^12^.

In the present study, we conducted a large-scale screening of secondary metabolites isolated from *Streptomyces* spp. and assessed their synergistic potential when combined with polymyxin B. The most effective secondary metabolite was identified as netropsin. Combination therapy consisting of polymyxin B and netropsin showed synergistic effects when tested on Gram-negative bacteria *in vitro*, including clinical isolates of colistin (polymyxin E)-resistant *A. baumannii*. The effectiveness of the combination therapy was verified *in vivo* using *G. mellonella* as a model host organism. Netropsin is a pyrrole-amide antitumor drug that binds preferentially to AT-rich regions in the minor groove of DNA and increases positive supercoiling^13^,^14^. Thus, the molecular specificity of netropsin makes it a versatile molecule for use not only against bacteria, but also potentially against viruses and tumors^15^.

## Results

### Development of novel screening system to identify antibiotics enhancing agents

**Figure 1:**
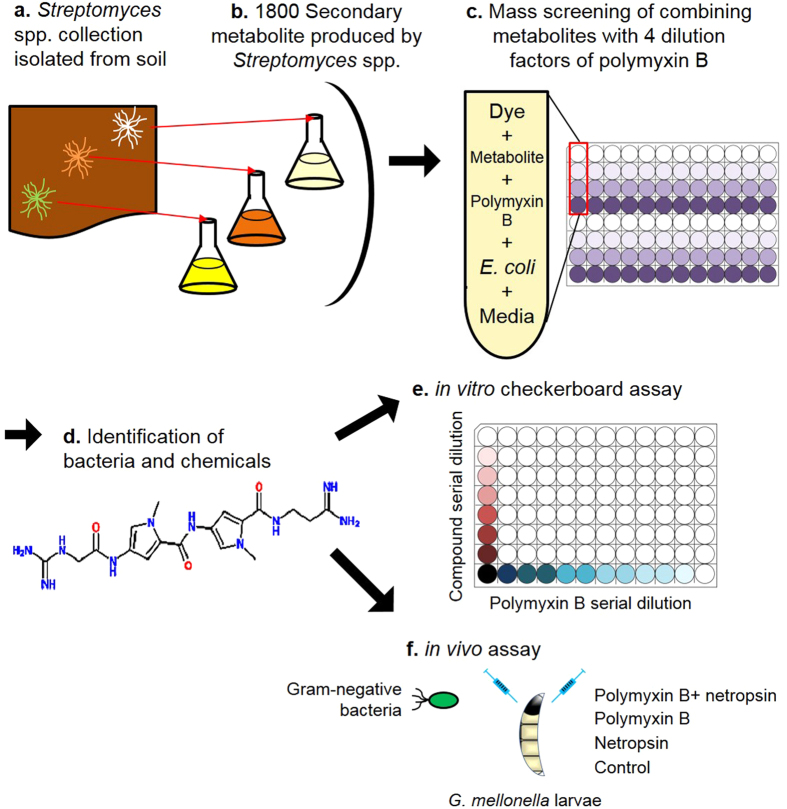
Large-scale screening strategy of secondary metabolites for mining agents that enhance the action of polymyxin B. A to F. Schematic representation of each experimental procedure to identify and characterize compounds that show synergism with polymyxin B. (**a**) Isolation of various *Streptomyces spp.* from different soil environmental samples. (**b**) Isolation of secondary metabolites from individual *Streptomyces spp.* (**c**) Mass screening of metabolites with serial concentrations of polmyxin B (250, 25, 2.5 and 0 mg/L), as indicated by violet color shades. Tetrazolium dye (0.5%) is included along with media, to measure the *E. coli* susceptibility to combined action of secondary metabolite and polymixin B. (**d**) Identification of synergetic compounds that originated from selected metabolite using 16 s rDNA sequencing and analytical chemistry techniques to identify compound within the metabolite responsible for synergism. (**e**) Checkerboard assay to test synergistic activity of compounds present in secondary metabolites against various Gram-negative pathogenic bacteria. (**f**) *In vivo* time kill assay using the greater wax moth to verify the effectiveness of combination therapy. Bacterial strains were infected to *G. mellonella* larvae followed by administration of polymixin B or netropsin or both.

Certain antibiotics such as polymixins are poor diffusing bactericidal agents on agar medium and hence difficult to obtain consistent results using traditional disc diffusion tests. Here we developed liquid media-based screening technique to screen for novel compounds that show synergetic effects with polymixin B. The strategy of mining antimicrobial agents from secondary metabolites library, their subsequent identification and evaluation both *in vitro* and *in vivo* is detailed in a step-by-step procedure (Fig. 1). **a.** Isolation of *Streptomyces* spp. from different soil environment. **b.** Construction of 1800 metabolite library from each isolated *Streptomyces* sp. **c.** Mass screening of metabolites with polmyxin B to test synergetic effects on *E. coli*. **d.** Identification of synergetic compound and bacteria producing the compound. **e.** *In vitro* checkerboard assay. **f.** Time kill assay using the greater wax moth infection model.

### Identification of compounds that enhance the antibiotic activity of polymixin B

To identify substances capable of enhancing the antibacterial action of polymyxin B, a strategy using a massive library of 1800 secondary metabolites isolated from equal number of *Streptomyces* spp. culture samples was established (Fig. 1a). A collection of 1800 strains of *Streptomyces* spp. were isolated from various soil samples that were obtained from forest, land, compost, and river water in S. Korea. Humic-vitamin medium was used for selective isolation of *Streptomyces* spp., which was then maintained as pure culture glycerol stocks. Glucose-Starch-Soybean (GSS) meal medium was used for activating secretory secondary metabolites produced from corresponding *Streptomyces* spp. culture stocks (Fig. 1b). Combinatorial effect of individual secondary metabolites with different concentrations of polymixin B was tested by measuring physiological change of *E. coli* oxidative metabolism as indicated by 0.5% tetrazolium dye (indicating oxygen consumption) (Fig. 1c). Of the extracts tested, an extract AN130070 showed the greatest synergistic effect at a minimum inhibitory concentration (MIC) of 0.25 mg/L of polymyxin B (Fig. S1). A checkerboard assay using serially diluted AN130070 metabolite with 0.25 mg/L polymixin B showed maximum synergy at zero dilution of AN130070 (Fig. S1). To identify the bacterial source of AN130070, genomic DNA of the corresponding stock was isolated and subjected to 16s rDNA sequencing. NCBI BLAST search of the sequence revealed that AN130070 metabolite was produced by *S. netropsis* SN01 (Fig. S2)^16^,^17^.

### Analysis of AN130070 by HPLC and MS

**Figure 2:**
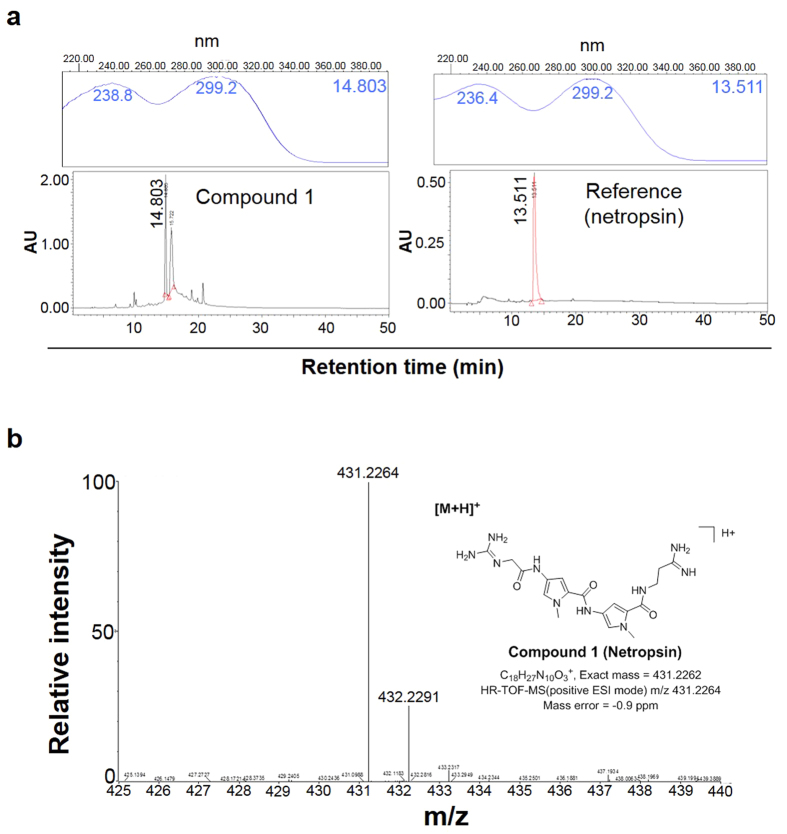
Identification of Netropsin. (**a**) HPLC spectrum of Compound 1 from AN130070 metabolite (left) and netropsin (right). Inner panel presented UV absorption of each peaks originated from subfraction of AN130070. RB625B12;Varian C18 column; 5~30% MeCN for 20 min, ~60% MeCN for 30 min, ~100% MeCN for 5 min, wash for 10 min, flow rate 1 mL/min, detection by absorption at 297 nm (**b**) Mass spectrum (positive ESI mode) of Compound 1 (Hao *et al.* 2013).

To identify the compounds responsible for the antibacterial activity of AN130070, a series of chemical fractionations were conducted. After each round, fractions were tested for synergy with polymyxin B to enhance bioactive fractions. Eight of 27 fractions (RB625B-12, 13, 19~24) were initially selected through C18HPLC to show synergism with polymyxin B. Compound 1 (RB625B-12A), obtained from RB625B-12 sub-fractions of AN130070 that is taken from the butanol layer exhibited synergistic effects with polymyxin B (Fig. S3). The Compound 1 was further analysed by HPLC to determine their chemical identity. UV absorption peak of the Compound 1 showed similar retention time compared with that of reference (commercial netropsin) (Fig. 2a). The ^1^H NMR inspection confirmed chemical structure of the Compound 1 compared with ^1^H NMR data of netropsin (Fig. S4)^18^. MS spectrum analysis of the Compound 1 from AN130070 extracted from *S. netropsis* SN01 showed two major peaks at m/z 431.2264 and 431.2291, and was consistent with the known spectrum for netropsin (Fig. 2b)^17^,^18^.

### Checkerboard assay for synergistic activity against Gram-negative bacteria

The MIC values of polymyxin B against several Gram-negative bacteria were lowered by adding netropsin, which indicated synergy. The bacteria tested included *E. coli* MG1655 and *A. baumannii* ATCC15150 that are a commensal bacterium and a human pathogen respectively. The fractional inhibitory concentration index (FICI) was then calculated to estimate the strength of the synergism. The FICI was 0.375 for *E. coli* MG1655 and 0.25 for *A. baumannii* ATCC15150, indicating strong synergism between netropsin and polymyxin B (Table 1). To test the practical application of a combination therapy using polymixin B and netropsin, a checkerboard assay was performed against clinical isolates of *A. baumannii* YCSAb5 and YCSAb7 that were obtained from Yonsei Severance Hospital, Seoul, S. Korea. For both isolates of *A. baumannii* YCSAb5 and YCSAb7, the FICI value was 0.375, showing synergism. In total 6 of 7 strains of *A. baumannii* showed FICI < 1.

Colistin (also referred to as polymyxin E) and polymyxin B are the same class peptide antibiotics and share similar structures and physicochemical properties. To test the combination of polymyxin B and netropsin against colistin-resistant, clinical isolates of *A. baumannii*, a checkerboard assay was carried out on five isolates. On colistin-resistant *A. baumannii* YCRAb257, the synergism was strong, with a FICI value of 0.266. Results with other isolates YCRAb 49, 269, 301 and 615 also indicated partial synergism of the polymyxin B and netropsin combination (Table 1).

The checkerboard assay was conducted for other Gram-negative bacteria- *S. flexneri* SF2a, *S. typhimurium* LT2, and *P. aeruginosa* PA14 and its clinical isolate YPa2. For *S. flexneri* SF2a and *S. typhimurium* LT2, there was partial synergism, with FICI values of 0.75 and 0.625, respectively (Table 1). When compared with the MICs of SF2a using polymyxin B alone and netropsin alone, the combination of netropsin with polymyxin B yielded 8- and 2-fold decrease in MICs, respectively. The FICI values for PA14 and YPa2 were 1 and 1.003, which indicated lack of synergism.

### Combination therapy using the greater wax moth, an alternative animal model

**Figure 3:**
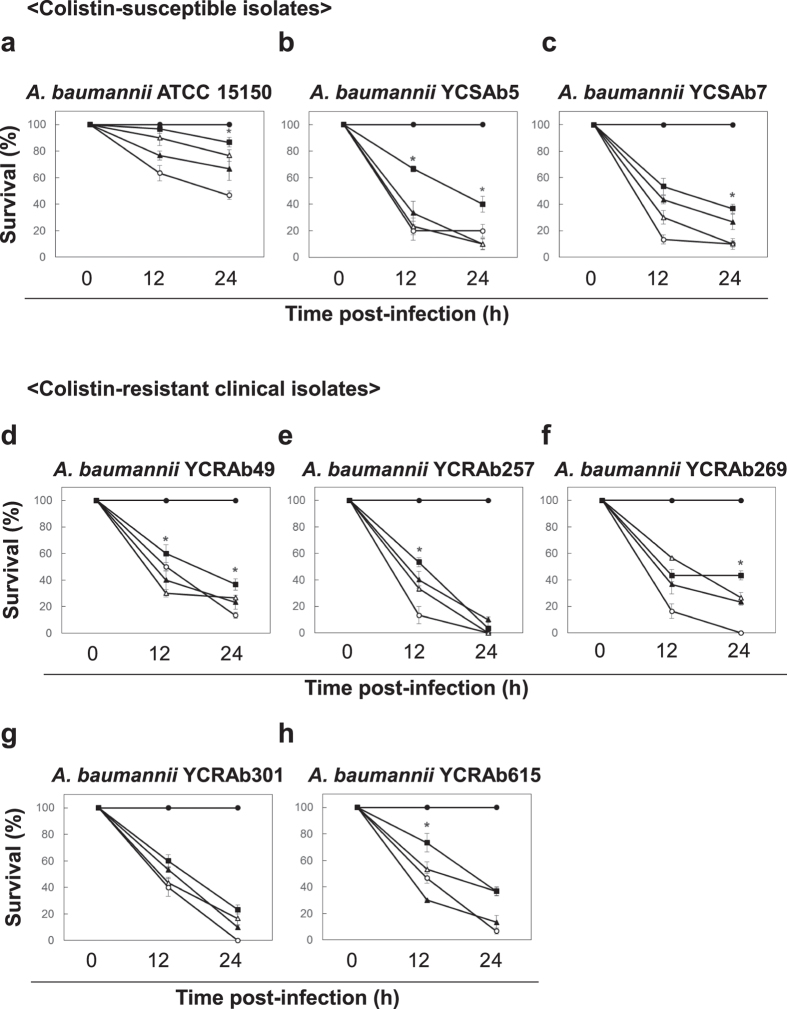
Synergistic effects of polymyxin B combination with netropsin against *A. baumannni* and its clinical isolates. The survival of *G. mellonella* infected with *A. baumannii*, its clinical isolates (YCSAb5 and YCSAb7), and colistin resistant clinical isolates (YCRAb49, 257, 269, 301, 615). (**a**) *A. baumanii* ATCC 15150, (**b**) YCSAb2, (**c**) YCSAb7, (**d**) YCRAb 49, (**e**) YCRAb 257, (**f**) YCRAb 269, (**g**) YCRAb 301, and (**h**) YCRAb 615. Treatments were: polymyxin B 4 mg/L (▲), netropsin 12.5 mg/L(△), polymyxin B 4 mg/L + netropsin 12.5 mg/L (■), control (○), and mock (●). Results represent means of three independent determinations ± standard deviations for 20 insects per treatment. An asterisk (*) indicates a significant difference between combination and monotherapy (*p* < 0.05).

We verified the results obtained above by performing a survival assay in *G. mellonella* infected with *A. baumannii* ATCC15150^19^. Polymyxin B and netropsin treatment concentrations were calculated from the MICs determined by *in vitro* assays. Concentrations of 0.5 mg/L of polymyxin B and 100 mg/L of netropsin were identified as the lowest active concentrations based on MIC values under *in vitro* conditions. The survival of *G. mellonella* was higher after 24 hours under treatment with the combination therapy (90%) than it was when polymyxin B (66.7%) or netropsin (76.67%) were administered individually. In addition, the survival of *G. mellonella* treated with the combination therapy was higher than that of untreated insects (46.7%; Fig. 3a).

Two clinical *A. baumannii* isolates YCSAb5 and YCSAb7 were tested *in vivo* for efficacy of combination therapy (Fig. 3b,c). The combination treatment exhibited potent synergism (YCSAb5, 40% and YCSAb7, 36.7%) as compared to polymyxin B (YCSAb5, 10% and YCSAb7, 26.7%) or netropsin (YCSAb5, 10% and YCSAb7, 10%) monotherapy 24 hours after treatment (Fig. 3b,c).

**Figure 4:**
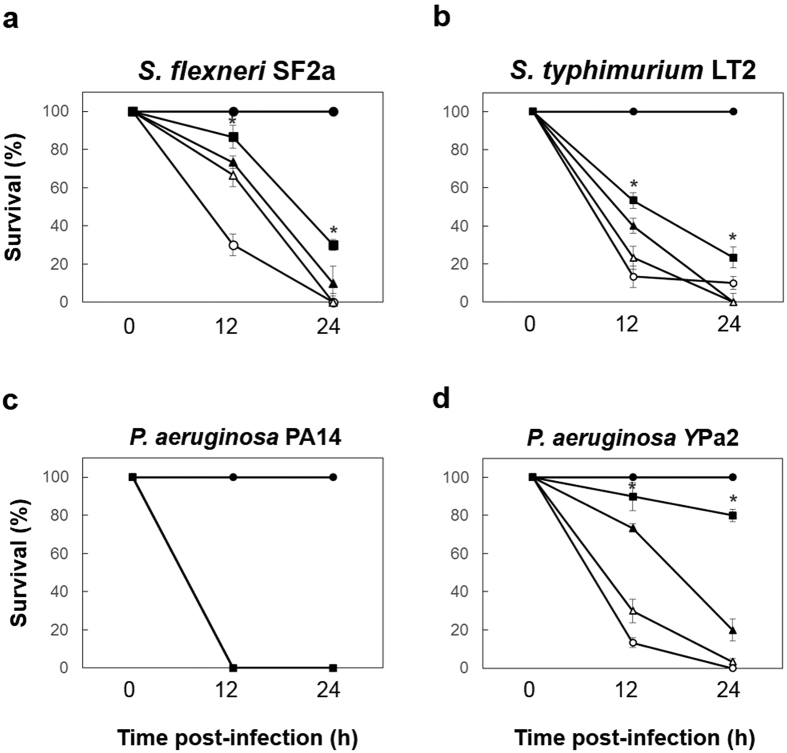
Synergistic effects of polymyxin B combination with netropsin. The survival of *G. mellonella* infected with (**a**) *S. flexneri* SF2a, (**b**) *S. typhimurium* LT2, (**c**) *P. aeruginosa* PA14 and (d) clinical isolates of *P. aeruginosa* YPa2 at 12 and 24 hour post-infection. Treatments were: polymyxin B 0.5 mg/L (▲), netropsin 100 mg/L (△), polymyxin B 0.5 mg/L + netropsin 100 mg/L (■), control (○), and mock (●). Results represent means of three independent determinations ± standard deviations for 20 insects per treatment. An asterisk (*) indicates a significant difference between combination and monotherapy (*p* < 0.05).

The combination therapy was also tested against five clinical isolates of colistin-resistant *A. baumannii*. We used the concentrations of single or double antibiotics to 4 mg/L of polymyxin B and 12.5 mg/L of netropsin based on Table 1. *A. baumannii* isolates YCRAb49, YCRAb257, and YCRAb615 showed relatively higher survival after 12 hours when treated with the combination therapy (YCRAb49, 60%; YCRAb257, 53.3%; and YCRAb615, 73.3%) than when treated with individual antibiotics (Fig. 4d,e,h). With YCRAb 269, the effects of the combination therapy lasted for 24 hours after treatment, and survival was higher (43.3%) than it was under monotherapy (polymyxin B, 23.3% and netropsin, 26.7%; Fig. 4f). However, the effect of the combination therapy on YCRAb 301 was not significantly different than that of monotherapy (Fig. 4g). A combination therapy using colistin and netropsin was also evaluated. In contrast to the combination of polymixin B and netropsin, the combination of colistin and netropsin did not exhibit a synergistic effect, except in the case of *A. baumannii* ATCC 15150 and YCRAb615 (Fig. S5).

Next, we tested the effectiveness of combination of polymyxin B and netropsin against other Gram-negative pathogenic bacteria (Fig. 4). The combination therapy resulted in 30% and 23.3% higher survival than monotherapy when used against *S. flxneri* SF2a (polymyxin B 10% and netropsin 0%) and *S. typhimurium* LT2 (polymyxin B 0% and netropsin 0%) infection, respectively (Fig. 4a,b). In the case of *P. aeruginosa* PA14 infection, there was no difference in survival under combination therapy (0%) compared to monotherapy or the control (0%; Fig. 4c). However the survival of *G. mellonella* inoculated with these clinical isolates was higher under treatment with combination therapy (80%) than when treated with monotherapy (polymyxin B 20% and netropsin 3.3%) or the untreated control (0%) in the case of *P. aerugnosa* YPa2 (Fig. 4d).

## Discussion

In this study, a novel combination therapy consisting of a last resort antibiotic (polymixin B) and an antitumor and antiviral drug (netropsin) is developed as an antimicrobial recipe against drug-resistance bacteria through *in vitro* screening of *Streptomyces* spp. secondary metabolites followed by *in vivo* evaluation (Fig. 1). Standard disc diffusion technique for polymixin susceptibility test is unreliable due to slow diffusion of polymixins on agar medium^20^. Here we utilized highly sensitive tetrazolium dye-based detection of synergism between natural compounds and polymixin B that allowed us to minimize antibiotic usage in the combination therapy. Bacterial secondary metabolites are the gold mine of antibiotics and are produced mainly by *Streptomyces* spp. in the idiophase^21^. We found that one of the metabolite among the screens; AN130070 is majorly composed of netropsin that markedly enhanced combinatorial effect with polymixin B against *E. coli*. Mono-treatment of AN130070 metabolite did not show significant inhibition, suggesting a potential adjuvant activity of netropsin (Fig. S1). The combination therapy was most effective against clinical isolates of *A. baumannii* wherein 6 out of 7 isolates tested showed FICI < 1 including colistin resistant isolates (Table 1). Presently colistin (polymyxin E) and polymixin B are the only two most clinically relevant polymixins with nearly identical spectrum of activity^20^. Despite similarities in the mechanism of action and toxicity between colistin and polymixin B, chemical structure, formulation, dosage and pharmacokinetic properties vary to a considerable extent between the two biochemical agents^22^. In our study, polymixin-netropsin combination therapy was effective against colistin resistant clinical isolates of *A. baumannii* with FICI < 0.5 (Table 1) and the colistin-netropsin therapy did not show same improvement, indicating that efficacy of combination therapy depends on formulation of type of polymixin used with netropsin (Figs 3 and S5). In addition, since the efficacy of colistin mono-therapy is low against colistin-resistant isolates, a combination therapy of colistin and netropsin can require further *in vivo* optimization that may vary from that of polymixin B-netropsin combination therapy^22^.

As such, role of netropsin has been primarily attributed to its antitumor and antiviral properties^23^. The crescent shaped molecule binds to the AT rich region of the DNA minor grove, thus inhibiting cellular processes such as replication, transcription and thus killing the aggressively dividing cancer cells rapidly^24^. The mechanism of action of netropsin has been extensively studied in the past^13^,^14^,^25^. Netropsin affects positive supercoiling of DNA and increases average twist per base but do not influence the contour length of the DNA *in vitro*^14^. However, some report suggests that netropsin alone do not significantly inhibit bacterial growth^26^. Of all the species tested, *A. baumannii* ATCC15150 showed highest synergy of FICI 0.25, while strains of *P. aeruginosa* showed FICI 1. The variations in the FICI values among different species tested are attributed to species-specific breakpoints of polymixin MIC values^20^. In particular, high efficacy against *A. baumannii* clinical isolates (FICI < 0.5) is of utmost clinical significance since polymyxin resistant *A. baumannii* are on a global rise at an alarming rate^3^,^4^. Overall, the decrease in the MIC of polymyxin B-netropsin combination therapy is statistically significant when compared to the reversed counterpart treatment.

The observed synergy was effective and consistent in the *G. mellonella* host. Few ethical constraints, a short life-cycle, and an innate immune response similar to that of higher vertebrates led us to use *G. mellonella* as an attractive model host^19^. Polymyxin B and netropsin combination therapy increased the survival of *G. mellonella* infected with *A. baumannii* upto 80% compared to survival without antibiotic treatment (Fig. 3). Monotherapies of polymixin B and netropsin showed lesser survival in most of the clinical isolates of *A. baumannii* and *P. aeruginosa* YPa2 compared to the combination therapy suggesting *in vivo* efficacy of the combination therapy (Figs 3b,c and 4d). Variation in survival number of individual positive controls of clinical isolates is reflected in the efficacy of the combination therapy. We did not see significant efficacy in case of *P. aeruginosa* Pa14 both *in vitro* and *in vivo* (Table 1, Fig. 4). In a pharmacokinetic study involving *P. aeruginosa* infection in *G. mellonella* larvae, little therapeutic benefit was seen treated with colistin^27^. Notably, there are no documented studies that show suitability of *G. mellonella* in studying combination therapies against Gram-negative bacteria. Treatment, optimization, and evaluation in clinically relevant model hosts are currently being explored to further develop the polymixin-netropsin combination therapy. Polymyxin B has been used in combination with numerous other antibiotics, such as carbapenems, efflux pump inhibitors, β-lactamase inhibitors, and wall teichoic acid synthesis inhibitors^10^. However, less clinical experience with polymixin B usage and resuscitation of polymixin resistant pathogenic strains demands more robust combination therapies, which minimize the use of multiple antibiotics^20^.

Off late polymixin B usage is increasingly promoted in the human health care. For example, a 3.5-year study extensively evaluated the clinical efficacy and safety profile of of polymixin B mono- and combination therapy for the respiratory tract infections caused by MDR *P. aeruginosa* and *A. baumannii*^28^. Of the 25 patients administered with 29 courses of polymixin B, 76% displayed favorable response while 52% survived the hospitalization with minimal nephro- and neurotoxcity. A total of 41% achieved microbiological clearance and only 21% displayed end-of-treatment mortality. Similarly more experiences that evaluate polymixin B with new adjuvants and therapeutic agents are required to minimize the dosage and maximize the effectiveness of the polymixin B combination therapy.

Presently, there are no DNA-binding, antibacterial agents qualified for clinical trial. Polymyxins have high affinity for lipopolysaccharides, as they competitively displace divalent cations from the bacteria’s outer membrane^20^,^29^. This allows transient uptake of a variety of molecules, leading to changes in membrane permeability and, ultimately, cell death^7^,^30^. Therefore, we speculate that polymyxin B enhances netropsin uptake through the outer membrane, thereby increasing the binding of netropsin to bacterial DNA and inhibiting cell growth^7^,^20^. However, this speculated model seems to have species-specific limitations. For example, synergy was not significant in case of *P. aeruginosa* both *in vitro* and *in vivo* (Table 1, Fig. 4) which could be attributed to highly evolved efflux pump systems and increased outer membrane impermeability that may render polymixin B and netropsin combination therapy ineffective^31^. Nevertheless, to the best of our knowledge, this is the first study to investigate *in vivo* efficacy of a DNA-binding antitumor agent with polymyxin B against Gram-negative bacteria that is demonstrated using the clinical MDR isolates of *A. baumannii.*

In conclusion, the combination therapy of polymxin B and netropsin provides a potential alternative to the existing limitations of polymixin usage and polymixin resistance. Mass screening of secondary metabolites isolated from *Streptomyces* spp. is a simple and effective method and hosts a plethora of useful compounds that are effective against a number of drug resistant, Gram-negative bacterial species including polymyxin B and colistin resistant *A. baumannii*. By using 96-well-containing tetrazolium dye-based *in vitro* high throughput screening and *G. mellonella*-based time kill studies we have demonstrated for the first time the efficacy of this novel combination therapy against polymixin B and colistin resistant clinical isolates of Gram-negative pathogenic bacteria.

## Methods

### Ethical considerations

Under the Korean Law of ‘Bioethics and Safety’, we had verbal approval from the hospital IRB committee to use the bacterial isolates from clinical samples without informed consents if we eliminated all the patients’ identifiers. The Committee approved all the experimental protocols using clinical isolates. All experimental procedures and methods were carried out in accordance with the approved protocols and the Committee’s guidelines.

### Bacterial strains and media

*E. coli* MG1655, *A. baumannii* ATCC15150, *P. aeruginosa* strain PA14, *Shigella flexneri* SF2a, and *Salmonella typhimurium* LT2 were grown in Luria-Bertani (LB) broth at 37 °C. Clinical specimens were isolated from blood, sputum, and faeces of patients suffering from *P. aeruginosa* YPa2, *A. baumanii* YCSAb5, and YCSAb7 infections. Clinical isolates of colistin-resistant *A. baumannii* YCRAb49, 257, 269, 301, and 615 were analysed in the hospital (Yonsei Severance Hospital, Seoul, S. Korea) and were grown at 37 °C in LB medium or agar (Asan Company) and Muller-Hinton agar (Asan Company)^32^,^33^.

### Construction of Streptomyces spp. secondary metabolite library

1800 of *Streptomyces* spp. were isolated from various soil samples that were obtained from forest, land, compost, and river water in S. Korea (http://www.mrscc.net). One gram wet weight of each soil sample was suspended in 9 ml of saline solution, serially diluted down to 10^−6^, and aliquots of each dilution (100 *μl*) was plated on humic-vitamin agar^34^, or fivefold-diluted R2A agar^35^, all supplemented with cycloheximide (50 *μ*g/ml) to reduce fungal contamination. Sample dilution plates were incubated at 28 °C for 14~21 days until sporulated or non-sporulated *Streptomyces* sp. colonies were observed and morphologically distinct colonies were isolated (Fig. 1a). A total of 1800 pure bacterial cultures were maintained on Bennett’s agar (10 g glucose, 1 g yeast extract, 2 g peptone, 1 g beef extract and 15 g of agar per liter; pH 7.0) and stored as 20% (v/v) glycerol suspensions at −80 °C. Individual bacterial samples were then inoculated into GSS liquid medium (10 g soluble starch, 20 g glucose, 25 g soybean meal, 1 g beef extract, 4 g yeast extract, 2 g NaCl, 0.25 g K_2_HPO_4_ and 2 g CaCO_3_ per liter; pH 7.2) to activate secondary metabolites secreted from correspondent *Streptomyces* sp. isolates and incubated at 28 °C with continuous shaking for 5–7 days at 140 rpm (Fig. 1b). Cultures were centrifuged at 10000 rpm and the supernatant was collected as the source of secretory secondary metabolite and stored at −4 °C for future use.

### Mass screening of secondary metabolites

A total of 50 μL of the individual secondary metabolites isolated from respective culture broths of *Streptomyces* spp. were added to 100 μL Muller-Hinton (MH) broth of minimal media containing 250, 25, 2.5, or 0 mg/L of polymyxin B and 0.5% tetrazolium dye as an indicator for oxidatively physiological change in individual wells of 96-well plates. After inoculation with 0.5% of a fresh culture of *E. coli* strain MG1655 (O.D._600_ = 0.3), the plates were incubated at 37 °C. The survival of MG1655 was determined by checking the colour change into violet (alive) or dead (transparent) and by measuring the OD_600 nm_ in a spectrophotometer (Fig. 1c). Selected metabolites were tested in triplicate. The checkerboard assay with polymyxin B and secreted metabolite from selected AN130070 was conducted to optimize the serial concentration of polymyxin B (1, 0.5, 0.25, 0.125, 0.625, 0.031, 0.016, and 0 mg/L) with serially diluted AN130070 metabolite (1, 0.5, 0.25, 0.13, 0.06, and 0; 5 dilution factors from initial concentration of AN130070 metabolite produced from *S. netropsis* SN01) (Fig. S1)^36^.

### Chemical fractionation of AN130070

A culture (10 L) of strain *S. netropsis* AN130070 was extracted three times with butanol (5.0 L). The extracts were combined and concentrated under reduced pressure to yield 19.0 g of dark brown gel. The gel was fractionated on a reversed-phase middle pressure liquid chromatography (MPLC) (Teledyne Isco, HP C18 Aq. Redisef Rf gold, 40 id × 300 mm; flow rate 7 mL/min, 100% H_2_O for 60 min, ~50% aq. MeOH for 300 min, MPLC 2. 50~100% MeOH for 300 min, wash for 60 min), yielding 27 sub-fractions. Sub-fractions 12 (120 mg, a brown gel) was purified by repeated, preparative C_18_ HPLC (Microsorb 100-5, C18, 21.4 id × 250 mm; gradient elution of 10 to 25% aqueous MeCN for 30 min, flow rate: 7 mL/min; detection by absorption at 297 nm) to afford Compound 1 (1.2 mg, t_R_ 33.0 min) (Figs 1d and S3).

### Identification of netropsin

NMR spectra were recorded on a Varian Mercury 400 spectrometer with standard pulse sequences operating at 400 MHz in ^1^H NMR. Chemical shifts measured in ppm, were referenced to solvent peak (δ_H_ 3.31 for CD_3_OD). Compound 1; ^1^H NMR(CD_3_OD, 400 MHz): δ 7.19 (1 H, s), 7.17 (1 H, s), 6.90 (1 H, s), 6.89 (1 H, s), 4.06 (2 H, s), 3.89 (3 H, s), 3.87 (3 H, s), 3.65 (2 H, m), 2.72 (2 H, s). High Resolution-ESI-MS spectra were recorded in the positive ESI (electrospray ionization) mode using a Waters SYNAPT G2 at the Korea Basic Science Institute. HR-TOF-MS (positive ESI mode) m/z 432.2264 [M + H]^+^ (calculated for C_18_H_27_N_10_O_3_^+^, 431.2262) (Fig. 1d).

### 16S rRNA gene sequencing

Genomic DNA from the strain was prepared using the method by Tamaoka and Komagata^37^. The 16S rRNA gene was amplified by PCR with the forward primer 27F and the reverse primer 1492R^16^. Direct sequence determination of the PCR amplified DNA was carried out using an automated DNA sequencer (model ABI 3730XL, Applied Biosystems). The 16S rRNA gene sequence of the strain was compared with available sequences from GenBank using the BLAST program (http://www.ncbi.nlm.nih.gov/BLAST/) to determine an approximate phylogenetic affiliation, and sequence similarity values were computed using the EzTaxon server (http://www.eztaxon.org/) (Fig. 1d)^38^.

### *In vitro* MIC tests

The checkerboard assay as *in vitro* assay was performed using commercially available netropsin in combination with polymyxin B (Fig. 1e). The initial concentrations of each antibiotic were determined from previously determined MIC_90_ values^39^,^40^,^41^. Wells containing the lowest fractional inhibitory concentrations were used to determine the fractional inhibitory concentration indices (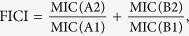 where A1 is the MIC value of antibiotic A monotherapy, A2 is the MIC value of antibiotic A combination therapy, B1 is the MIC value of antibiotic B monotherapy, and B2 is the MIC value of antibiotic B combination therapy). Synergistic effects were defined in terms of FICI values as ≤0.5 (perfect synergy), >0.5 to <1 (partial synergy), a value of 1 (additive effect), and >1 (loss of synergism). MIC values were interpreted as previously described^36^,^42^. MIC_pol/net_ range value were determined the series of concentration of first-indicated antibiotics (polymyxin) concentration range in the presence of the second-indicated antibiotics (netropsin) showing synergism in the checkerboard assay in Table 1 (opposite to MIC_net/pol_)^9^.

### *G. mellonella* time kill assay

To confirm the synergism of polymyxin B with netropsin *in vivo*, time kill assay of greater wax moth, *Galleria mellonella,* as a model system were carried out (Fig. 1f). *G. mellonella* caterpillars were grown at 37 °C until they reached the fourth larval instar (Ecowin). Caterpillars were then stored in the dark at 37 °C for 3 days for stabilization. Bacterial cells were washed with phosphate-buffered saline and diluted to 10^6^ cfu/larva. Bacterial inocula were injected into the left and right abdominal cavities of 20 randomly selected larvae using 5 μL micro-syringes (Microliter^TM^ #701, Hamilton). Within 30 min of infection with the microbial suspension, monotherapy or combination therapy was performed on the caterpillars by injecting antibiotic into different prolegs. The amount of antibiotic (polymyxin B and netropsin, Sigma) administered to individual caterpillars was determined by larval weight and MIC results from the *in vitro* assay. Infected caterpillars were kept in a 37 °C incubator to assess survival 12 and 24 hours after inoculation^43^. All experiments were repeated thrice.

### Data analysis

All experiments were repeated thrice. The results were reported as the mean ± SD from at least three representative experiments. Statistical analyses were carried out with JMPIN 4.0.4 (SAS Institute) using one-way ANOVA followed by a Student’s t-test for treatment comparisons. Differences were considered to be significant at *p* < 0.05.

## Additional Information

**How to cite this article**: Chung, J.-h. *et al.* Combination therapy with polymyxin B and netropsin against clinical isolates of multidrug-resistant *Acinetobacter baumannii. Sci. Rep.* **6**, 28168; doi: 10.1038/srep28168 (2016).

## Supplementary Material

Supplementary Information

## Figures and Tables

**Table 1:** *In vitro* MIC and FICI values for treatments against Gram-negative bacterial strains.

Species	FICI_polB/net_ Range	MIC_polB_ (A1)	Com_polB_(A2)	MIC_polB/net_ Range	MIC_net_ (B1)	Com_net_(B2)	MIC_net/polB_ Range
*A. baumanii*, clinical isolates (YCSAb5, 7), and colistin resitant clinical isolates (YCRAb49, 257, 269, 301, 615)							
*A. baumannii* ATCC15150	0.25	2	0.25	0.001953–0.5	200	25	3.125–100
*A. baumanii* YCSAb5	0.375	1	0.25	0.125–0.25	200	25	3.2–100
*A. baumanii* YCSAb7	0.375	2	0.25	0.125–1	200	50	12.5–100
*A. baumanii* YCRAb49	0.75	16	8	8	800	200	200–400
*A. baumanii* YCRAb257	0.266	16	4	0.125–2	200	3.125	12.5–100
*A. baumanii* YCRAb269	0.508	8	4	0.25–2	400	3.125	3.125–100
*A. baumanii* YCRAb301	0.508	8	4	0.5–2	400	3.125	3.125–200
*A. baumanii* YCRAb615	0.508	8	4	0.125–2	400	3.125	3.125–200
Commensal and human pathogenic Gram-negative bacteria							
*E. coli* MG1655	0.375	1	0.125	0.0625–0.125	50	12.5	0.098–25
*S. flexneri* SF2a	0.75	1	0.25	0.125–0.5	12.5	6.25	3.125–6.25
*S. typhimurium* LT2	0.625	4	0.5	0.250–0.5	25	12.5	6.25–12.5
*P. aeruginosa* PA14	1	1	0.5	0.5–1	400	200	400–200
*P. aeruginosa* YPa2	1.004	2	2	2	800	3.125	800

Abbreviations: Com, the most inhibitory concentration (MIC) value of combined antibiotics; FICI, fractional inhibitory concentration index; FICI_polB/net_ indicates the values at the most synergistic combination of polymyxin B and netropsin; MIC, minimum inhibitory concentration; net, netropsin; polB, polymyxin B; MIC_polB/net_ and MIC_net/polB_ represent the series of MIC values of polymyxin B or netropsin in the presence of netropsin or polymyxin B except the MIC values of monotherapy, respectively. FICI = MIC(A2)/MIC(A1) + MIC(B2)/MIC(B1). All MIC values are in mg/L.
