# Supplementary material for: Combination therapy with polymyxin B and netropsin against clinical isolates of multidrug-resistant Acinetobacter baumannii
Source: Sci Rep. 2016 Jun 16;6:28168. doi: 10.1038/srep28168 (PMC4910107; doi:10.1038/srep28168)
Supplement: Supplementary Information [file srep28168-s1.pdf]

Combination therapy with polymyxin B and netropsin against clinical isolates of multidrug-resistant *Acinetobacter baumannii*

Joon-hui Chung[1,2], Abhayprasad Bhat[1], Chang-Jin Kim[1,2], Dongeun Yong[3] & Choong-Min Ryu[1,2]

[1]Super-bacteria Research Center, Korea Research Institute of Bioscience and Biotechnology, Daejeon 34141, South Korea;

[2]University of Science and Technology, Daejeon 34113, South Korea;

[3]Industrial Bio-materials Research Center, Korea Research Institute of Bioscience and Biotechnology, Daejeon 34141, South Korea;

[4]Department of Laboratory Medicine and Research Institute of Antimicrobial Resistance, Yonsei University College of Medicine, Seoul 03722, South Korea. Correspondence and requests for materials should be addressed to C.-M. R. (email: cmryu@kribb.re.kr)

Supplementary figure S1. (Upper) Screening results from combination polymyxin B (0.250 and 0 mg/L) and AN130070 of *Streptomyces netropsis* SN01 metabolite using colour change of 0.5% tetrazolium dye. *E. coli* MG1655 (O.D.^{600} = 0.3) was inoculated at tetrazolium-containing 96-well plates with polymyxin B and secondary metabolites (Lower) Checkerboard assay of polymyxin B with AN130070 metabolite. AN130070 and polymyxin B were serially diluted on 96-well plates.

Supplementary figure S2. Sequence alignment of AN130070 16s rDNA with a reference sequence of *S. netropsis* HBUM83782 16s rDNA. Query and subject refers to the AN130070 rDNA sequence and the reference sequence, respectively.

Supplementary figure S3. Flowchart for fractionation of AN130070. The gel concentrated from AN130070 metabolite was fractionated on a reversed-phase middle pressure liquid chromatography (MPLC) (Teledyne Isco, HP C18 Aq. Redisef Rf gold, 40 id x 300 mm; flow rate 7 mL/min, 100% H_2O for 60 min, ~50% aq. MeOH for 300 min, MPLC 2. 50~100% MeOH for 300 min, wash for 60 min), yielding 27 sub-fractions. Sub-fractions 12 (120 mg, a brown gel) was purified by repeated, preparative C18 HPLC (Microsorb 100-5, C18, 21.4 id x250 mm; gradient elution of 10 to 25% aqueous MeCN for 30 min, flow rate: 7 mL/min; detection by absorption at 297 nm) to afford Compound 1 (1.2 mg, tR 33.0 min)

Supplementary figure S4. **^{1}H NMR spectrum of Compound 1.** NMR spectra were recorded on a Varian Mercury 400 spectrometer with standard pulse sequences operating at 400 MHz in 1H NMR. Chemical shifts measured in ppm, were referenced to solvent peak (δH 3.31 for CD3OD).

Supplementary figure S5. Synergistic effects of colistin-netropsin combination therapy. The survival of *G. mellonella* infected with *A. baumannii* and its colistin resistant clinical isolates. (**a**) *A. baumannii* ATCC 15150, (**b**) YCRAb 49, (**c**) YCRAb 257, (**d**) YCRAb 269, (**e**) YCRAb 301, and (**f**) YCRAb 615. Treatments were: colistin 4 mg/L (▲), netropsin 12.5 mg/L(△), colistin 4 mg/L + netropsin 12.5 mg/L (■), control (○), and mock (●). Results represent means of three independent determinations ± standard deviations for 20 insects per treatment. An asterisk (*) indicates a significant difference between combination and monotherapy ($p < 0.05$).

Fig. S1

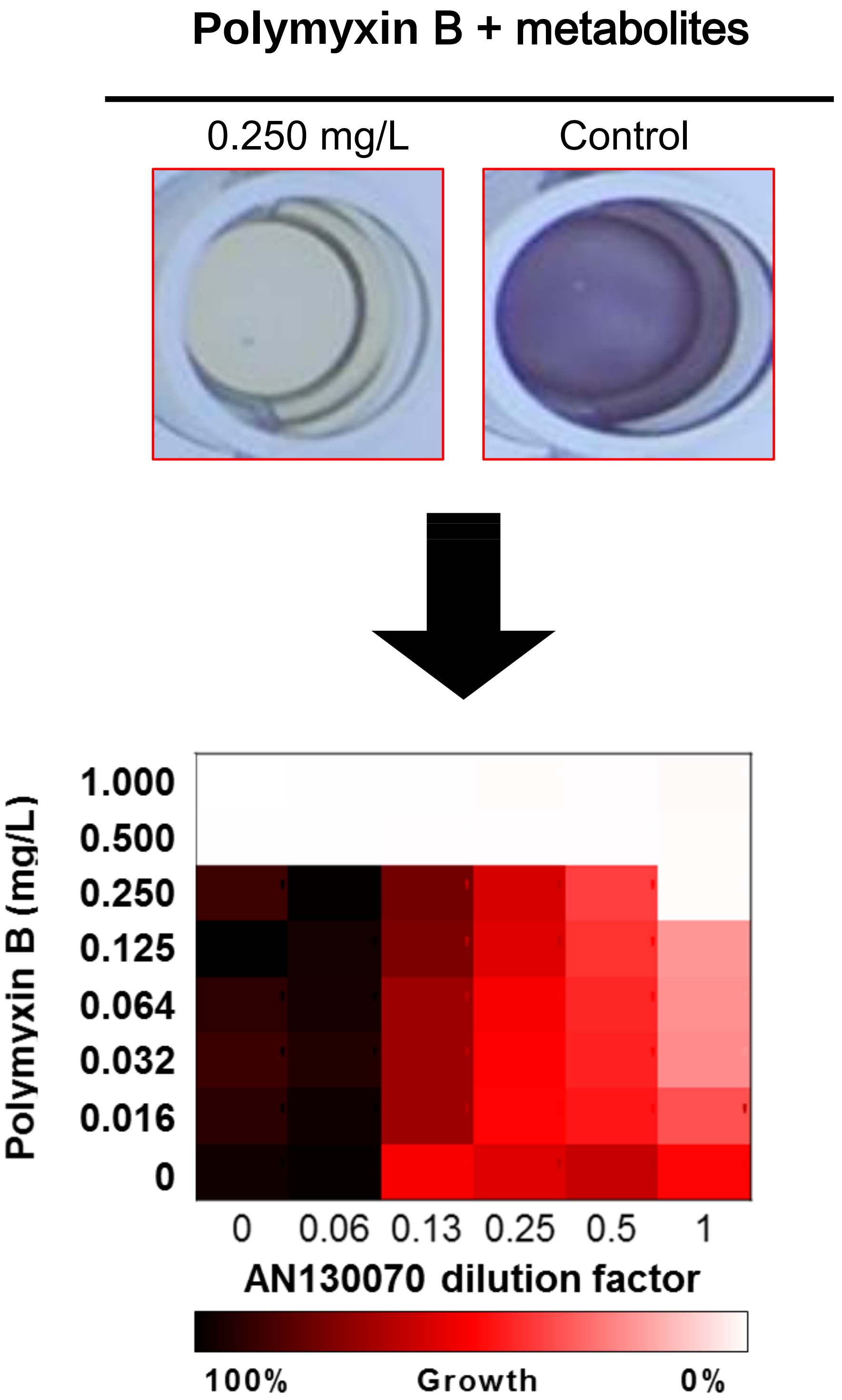
Polymyxin B + metabolites
0.250 mg/L
Control
Polymyxin B (mg/L)
1.000
0.500
0.250
0.125
0.064
0.032
0.016
0
0
0.06
0.13
0.25
0.5
1
AN130070 dilution factor
100%
Growth
0%

Fig. S2

Streptomyces netropsis strain HBUM83782 16S ribosomal RNA gene, partial sequence

Sequence ID: gb|EU841571.1| Length: 1437 Number of Matches: 1

Range 1: 13 to 1383 GenBank Graphics ▼ Next Match ▲ Previous Match

Score	Expect	Identities	Gaps	Strand
2525 bits(1367)	0.0	1370/1371(99%)	1/1371(0%)	Plus/Plus

```
Query  1     AAGTCGAACGATGAAGCCTTTCGGGGTGGATTAGTGGCGAACGGGTGAGTAACACGTGGG  60
             ||||||||||||||||||||||||||||||||||||||||||||||||||||||||||||
Sbjct  13    AAGTCGAACGATGAAGCCTTTCGGGGTGGATTAGTGGCGAACGGGTGAGTAACACGTGGG  72

Query  61    CAATCTGCCCTTCACTCTGGGACAAGCCCTGGAAACGGGGTCTAATACCGGATACGACCT  120
             ||||||||||||||||||||||||||||||||||||||||||||||||||||||||||||
Sbjct  73    CAATCTGCCCTTCACTCTGGGACAAGCCCTGGAAACGGGGTCTAATACCGGATACGACCT  132

Query  121   GCCTCCGCATGGGGGTGGGTGGAAAGCTCCGGCGGTGAAGGATGAGCCCGCGGCCTATCA  180
             ||||||||||||||||||||||||||||||||||||||||||||||||||||||||||||
Sbjct  133   GCCTCCGCATGGGGGTGGGTGGAAAGCTCCGGCGGTGAAGGATGAGCCCGCGGCCTATCA  192

Query  181   GCTTGTTGGTGGGGTAATGGCCTACCAAGGCGACGACGGGTAGCCGGCCTGAGAGGGCGA  240
             ||||||||||||||||||||||||||||||||||||||||||||||||||||||||||||
Sbjct  193   GCTTGTTGGTGGGGTAATGGCCTACCAAGGCGACGACGGGTAGCCGGCCTGAGAGGGCGA  252

Query  241   CCGGCCACACTGGGACTGAGACACGGCCCAGACTCCTACGGGAGGCAGCAGTGGGGAATA  300
             ||||||||||||||||||||||||||||||||||||||||||||||||||||||||||||
Sbjct  253   CCGGCCACACTGGGACTGAGACACGGCCCAGACTCCTACGGGAGGCAGCAGTGGGGAATA  312

Query  301   TTGCACAATGGGCGAAAGCCTGATGCAGCGACGCCGCGTGAGGGATGACGGCCTTCGGGT  360
             ||||||||||||||||||||||||||||||||||||||||||||||||||||||||||||
Sbjct  313   TTGCACAATGGGCGAAAGCCTGATGCAGCGACGCCGCGTGAGGGATGACGGCCTTCGGGT  372

Query  361   TGTAAACCTCTTTCAGCAGGGAAGAAGCGAGAGTGACGGTACCTGCAGAAGAAGCGCCGG  420
             ||||||||||||||||||||||||||||||||||||||||||||||||||||||||||||
Sbjct  373   TGTAAACCTCTTTCAGCAGGGAAGAAGCGAGAGTGACGGTACCTGCAGAAGAAGCGCCGG  432

Query  421   CTAACTACGTGCCAGCAGCCGCGGTAATACGTAGGGCGCAAGCGTTGTCCGGAATTATTG  480
             ||||||||||||||||||||||||||||||||||||||||||||||||||||||||||||
Sbjct  433   CTAACTACGTGCCAGCAGCCGCGGTAATACGTAGGGCGCAAGCGTTGTCCGGAATTATTG  492

Query  481   GGCGTAAAGAGCTCGTAGGCGGCTTGTTGCGTCGGATGTGAAAGCCCGGGGCTTAACCCC  540
             ||||||||||||||||||||||||||||||||||||||||||||||||||||||||||||
Sbjct  493   GGCGTAAAGAGCTCGTAGGCGGCTTGTTGCGTCGGATGTGAAAGCCCGGGGCTTAACCCC  552

Query  541   GGGTCTGCATTCGATACGGGCAGGCTAGAGTGTGGTAGGGGAGATCGGAATTCCTGGTGT  600
             ||||||||||||||||||||||||||||||||||||||||||||||||||||||||||||
Sbjct  553   GGGTCTGCATTCGATACGGGCAGGCTAGAGTGTGGTAGGGGAGATCGGAATTCCTGGTGT  612

Query  601   AGCGGTGAAATGCGCAGATATCAGGAGGAACACCGGTGGCGAAGGCGGATCTCTGGGCCA  660
             ||||||||||||||||||||||||||||||||||||||||||||||||||||||||||||
Sbjct  613   AGCGGTGAAATGCGCAGATATCAGGAGGAACACCGGTGGCGAAGGCGGATCTCTGGGCCA  672

Query  661   TTACTGACGCTGAGGAGCGAAAGCGTGGGGAGCGAACAGGATTAGATACCCTGGTAGTCC  720
             ||||||||||||||||||||||||||||||||||||||||||||||||||||||||||||
Sbjct  673   TTACTGACGCTGAGGAGCGAAAGCGTGGGGAGCGAACAGGATTAGATACCCTGGTAGTCC  732

Query  721   ACGCCGTAAACGTTGGGAACTAGGTGTTGGCGACATTCCACGTCGTCGGTGCCGCAGCTA  780
             ||||||||||||||||||||||||||||||||||||||||||||||||||||||||||||
Sbjct  733   ACGCCGTAAACGTTGGGAACTAGGTGTTGGCGACATTCCACGTCGTCGGTGCCGCAGCTA  792

Query  781   ACGCATTAAGTTCCCCGCCTGGGGAGTACGGCCGCAAGGCTAAAACTCAAAGGAATTGAC  840
             ||||||||||||||||||||||||||||||||||||||||||||||||||||||||||||
Sbjct  793   ACGCATTAAGTTCCCCGCCTGGGGAGTACGGCCGCAAGGCTAAAACTCAAAGGAATTGAC  852

Query  841   GGGGGCCCGCACAAGCAGCGGAGCATGTGGCTTAATTCGACGCAACGCGAAGAACCTTAC  900
             ||||||||||||||||||||||||||||||||||||||||||||||||||||||||||||
Sbjct  853   GGGGGCCCGCACAAGCAGCGGAGCATGTGGCTTAATTCGACGCAACGCGAAGAACCTTAC  912

Query  901   CAAGGCTTGACATATACCGGAAACGGCCAGAGATGGTCGCCCCCTTGTGGTCGGTATACA  960
             ||||||||||||||||||||||||||||||||||||||||||||||||||||||||||||
Sbjct  913   CAAGGCTTGACATATACCGGAAACGGCCAGAGATGGTCGCCCCCTTGTGGTCGGTATACA  972

Query  961   GGTGGTGCATGGCTGTCGTCAGCTCGTGTCGTGAGATGTTGGGTTAAGTCCCGCAACGAG  1020
             ||||||||||||||||||||||||||||||||||||||||||||||||||||||||||||
Sbjct  973   GGTGGTGCATGGCTGTCGTCAGCTCGTGTCGTGAGATGTTGGGTTAAGTCCCGCAACGAG  1032

Query  1021  CGCAACCCTTGTTCTGTGTTGCCAGCATGCCTTTCGGGGTGATGGGGACTCACAGGAGAC  1080
             ||||||||||||||||||||||||||||||||||||||||||||||||||||||||||||
Sbjct  1033  CGCAACCCTTGTTCTGTGTTGCCAGCATGCCTTTCGGGGTGATGGGGACTCACAGGAGAC  1092

Query  1081  TGCCGGGGTCAACTCGGAGGAAGGTGGGGACGACGTCAAGTCATCATGCCCCTTATGTCT  1140
             ||||||||||||||||||||||||||||||||||||||||||||||||||||||||||||
Sbjct  1093  TGCCGGGGTCAACTCGGAGGAAGGTGGGGACGACGTCAAGTCATCATGCCCCTTATGTCT  1152

Query  1141  TGGGCTGCACACGTGCTACAATGGCCGGTACAATGAGCTGCGATACCGTGAGGTGGAGCG  1200
             ||||||||||||||||||||||||||||||||||||||||||||||||||||||||||||
Sbjct  1153  TGGGCTGCACACGTGCTACAATGGCCGGTACAATGAGCTGCGATACCGTGAGGTGGAGCG  1212

Query  1201  AATCTCAAAAAGCCGGTCTCAGTTCGGATTGGGGTCTGCAACTCGACCCCATGAAGTTGG  1260
             ||||||||||||||||||||||||||||||||||||||||||||||||||||||||||||
Sbjct  1213  AATCTCAAAAAGCCGGTCTCAGTTCGGATTGGGGTCTGCAACTCGACCCCATGAAGTTGG  1272

Query  1261  AGTTGCTAGTAATCGCAGATCAGCATTGCTGCGGTGAATACGTTCCCGGGCCTTGTACAC  1320
             ||||||||||||||||||||||||||||||||||||||||||||||||||||||||||||
Sbjct  1273  AGTTGCTAGTAATCGCAGATCAGCATTGCTGCGGTGAATACGTTCCCGGGCCTTGTACAC  1332

Query  1321  ACCGCCCGTCACGTCACGAAAGTCGGTAACACCCGAAGCCGGTG-CCCAAC  1370
             |||||||||||||||||||||||||||||||||||||||||||| ||||||
Sbjct  1333  ACCGCCCGTCACGTCACGAAAGTCGGTAACACCCGAAGCCGGTGGCCCAAC  1383
```

Fig. S3

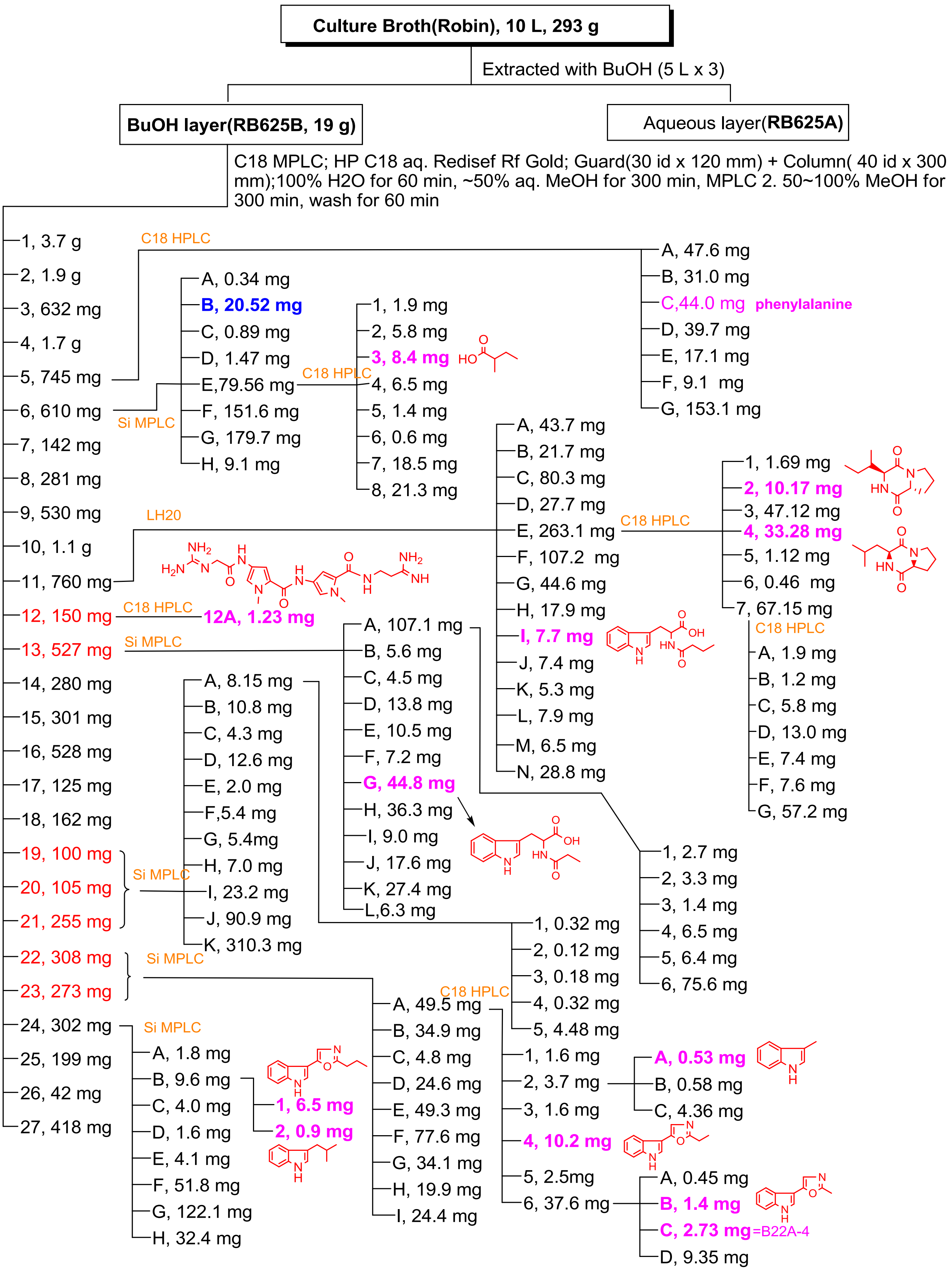

Culture Broth(Robin), 10 L, 293 g
Extracted with BuOH (5 L x 3)
BuOH layer(RB625B, 19 g)
Aqueous layer(RB625A)
C18 MPLC; HP C18 aq. Redisef Rf Gold; Guard(30 id x 120 mm) + Column(40 id x 300 mm);100% H2O for 60 min, ~50% aq. MeOH for 300 min, MPLC 2. 50~100% MeOH for 300 min, wash for 60 min
1, 3.7 g
2, 1.9 g
3, 632 mg
4, 1.7 g
5, 745 mg
6, 610 mg
7, 142 mg
8, 281 mg
9, 530 mg
10, 1.1 g
11, 760 mg
12, 150 mg
13, 527 mg
14, 280 mg
15, 301 mg
16, 528 mg
17, 125 mg
18, 162 mg
19, 100 mg
20, 105 mg
21, 255 mg
22, 308 mg
23, 273 mg
24, 302 mg
25, 199 mg
26, 42 mg
27, 418 mg
C18 HPLC
A, 47.6 mg
B, 31.0 mg
C,44.0 mg phenylalanine
D, 39.7 mg
E, 17.1 mg
F, 9.1 mg
G, 153.1 mg
Si MPLC
A, 0.34 mg
B, 20.52 mg
C, 0.89 mg
D, 1.47 mg
E,79.56 mg
F, 151.6 mg
G, 179.7 mg
H, 9.1 mg
C18 HPLC
1, 1.9 mg
2, 5.8 mg
3, 8.4 mg
4, 6.5 mg
5, 1.4 mg
6, 0.6 mg
7, 18.5 mg
8, 21.3 mg
LH20
A, 43.7 mg
B, 21.7 mg
C, 80.3 mg
D, 27.7 mg
E, 263.1 mg
F, 107.2 mg
G, 44.6 mg
H, 17.9 mg
I, 7.7 mg
J, 7.4 mg
K, 5.3 mg
L, 7.9 mg
M, 6.5 mg
N, 28.8 mg
C18 HPLC
1, 1.69 mg
2, 10.17 mg
3, 47.12 mg
4, 33.28 mg
5, 1.12 mg
6, 0.46 mg
7, 67.15 mg
C18 HPLC
A, 1.9 mg
B, 1.2 mg
C, 5.8 mg
D, 13.0 mg
E, 7.4 mg
F, 7.6 mg
G, 57.2 mg
C18 HPLC
12A, 1.23 mg
Si MPLC
A, 107.1 mg
B, 5.6 mg
C, 4.5 mg
D, 13.8 mg
E, 10.5 mg
F, 7.2 mg
G, 44.8 mg
H, 36.3 mg
I, 9.0 mg
J, 17.6 mg
K, 27.4 mg
L,6.3 mg
1, 2.7 mg
2, 3.3 mg
3, 1.4 mg
4, 6.5 mg
5, 6.4 mg
6, 75.6 mg
Si MPLC
A, 8.15 mg
B, 10.8 mg
C, 4.3 mg
D, 12.6 mg
E, 2.0 mg
F,5.4 mg
G, 5.4mg
H, 7.0 mg
I, 23.2 mg
J, 90.9 mg
K, 310.3 mg
1, 0.32 mg
2, 0.12 mg
3, 0.18 mg
4, 0.32 mg
5, 4.48 mg
Si MPLC
C18 HPLC
A, 49.5 mg
B, 34.9 mg
C, 4.8 mg
D, 24.6 mg
E, 49.3 mg
F, 77.6 mg
G, 34.1 mg
H, 19.9 mg
I, 24.4 mg
1, 1.6 mg
2, 3.7 mg
3, 1.6 mg
4, 10.2 mg
5, 2.5mg
6, 37.6 mg
A, 0.53 mg
B, 0.58 mg
C, 4.36 mg
A, 0.45 mg
B, 1.4 mg
C, 2.73 mg =B22A-4
D, 9.35 mg
Si MPLC
A, 1.8 mg
B, 9.6 mg
C, 4.0 mg
D, 1.6 mg
E, 4.1 mg
F, 51.8 mg
G, 122.1 mg
H, 32.4 mg
1, 6.5 mg
2, 0.9 mg

Fig. S4

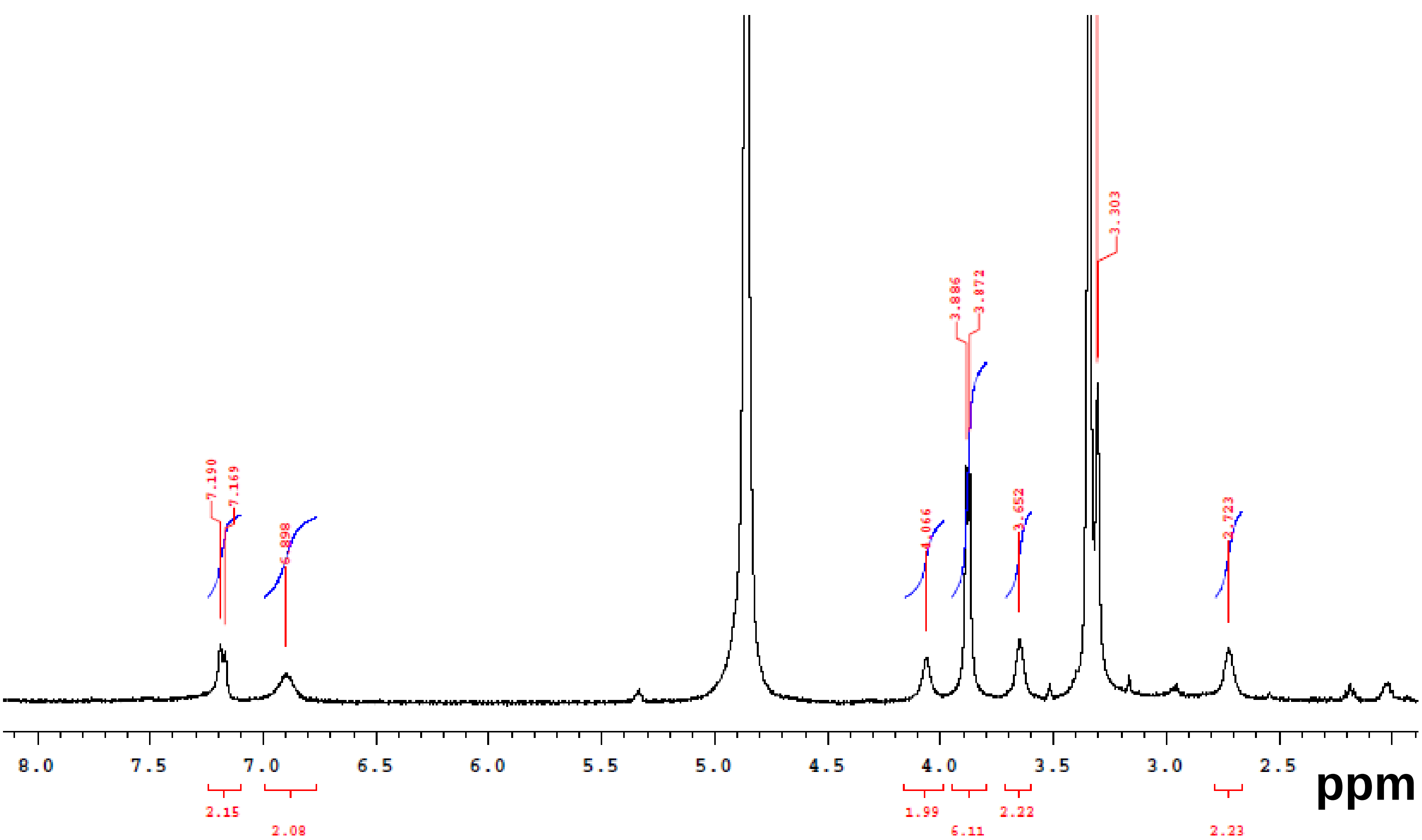

7.190
7.169
6.898
4.066
3.886
3.872
3.652
3.303
2.723
8.0
7.5
7.0
6.5
6.0
5.5
5.0
4.5
4.0
3.5
3.0
2.5
ppm
2.15
2.08
1.99
6.11
2.22
2.23

Fig. S5

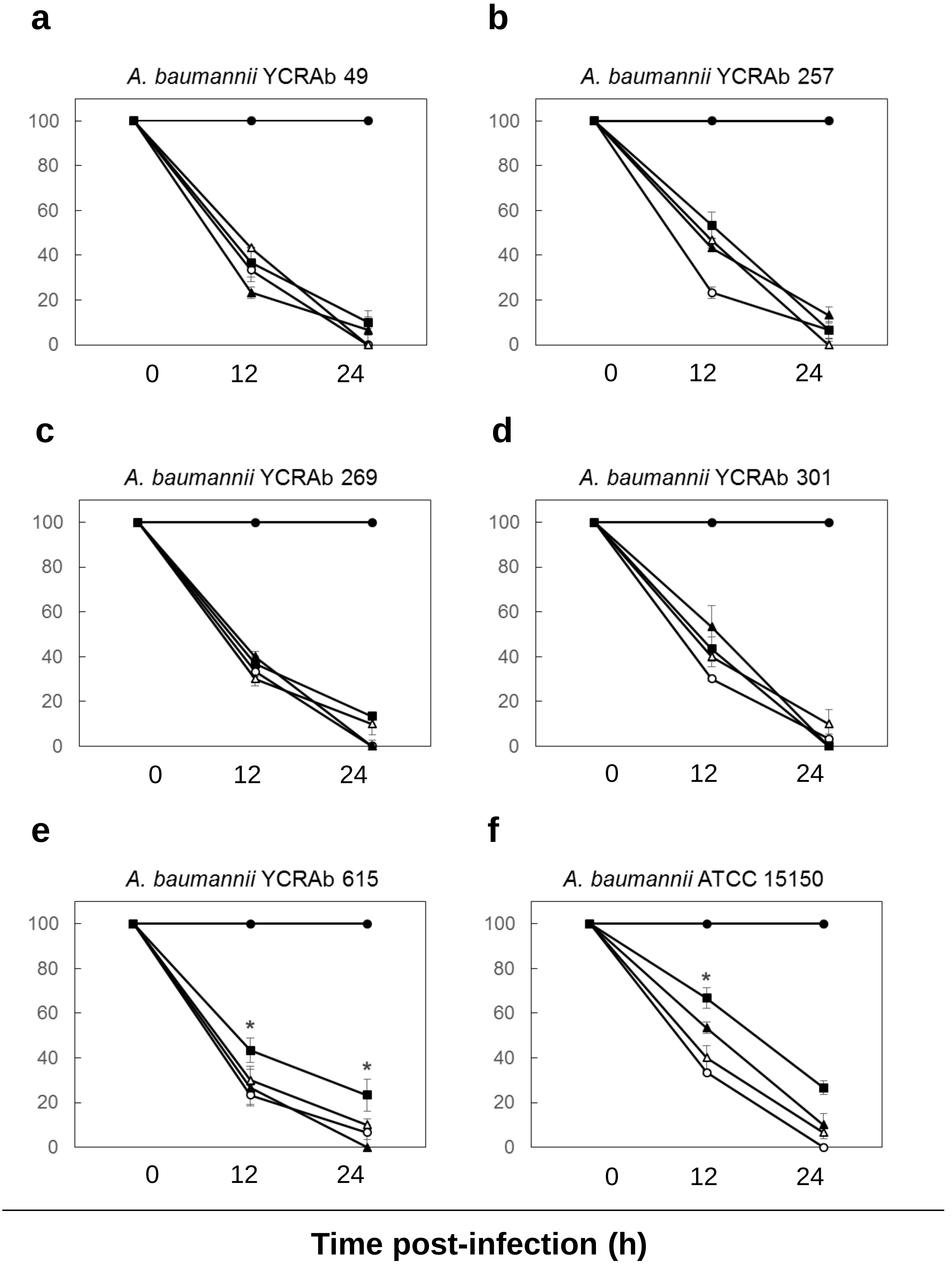

a
A. baumannii YCRAb 49
b
A. baumannii YCRAb 257
c
A. baumannii YCRAb 269
d
A. baumannii YCRAb 301
e
A. baumannii YCRAb 615
f
A. baumannii ATCC 15150
100
80
60
40
20
0
0
12
24
*
Time post-infection (h)
